# Identifying common and specific microRNAs expressed in peripheral blood mononuclear cell of type 1, type 2, and gestational diabetes mellitus patients

**DOI:** 10.1186/1756-0500-6-491

**Published:** 2013-11-26

**Authors:** Cristhianna VA Collares, Adriane F Evangelista, Danilo J Xavier, Diane M Rassi, Thais Arns, Maria C Foss-Freitas, Milton C Foss, Denis Puthier, Elza T Sakamoto-Hojo, Geraldo A Passos, Eduardo A Donadi

**Affiliations:** 1 Department of Medicine, Division of Clinical Immunology, Faculty of Medicine of Ribeirao Preto, University of São Paulo, 14048-900 Ribeirao Preto, SP, Brazil; 2 Department of Genetics, Molecular Immunogenetics Group, Faculty of Medicine of Ribeirao Preto, University of São Paulo, 14040-900 Ribeirao Preto, SP, Brazil; 3 Department of Medicine, Division of Endocrinology, Faculty of Medicine of Ribeirao Preto, University of São Paulo, 14048-900 Ribeirao Preto, SP, Brazil; 4 Aix-Marseille University, Marseille, France; 5 Faculty of Philosophy Sciences and Letters of Ribeirao Preto, University of Sao Paulo, Ribeirao Preto, SP, Brazil

**Keywords:** Type 1 diabetes mellitus, Type 2 diabetes mellitus, Gestational diabetes mellitus, microRNA, mRNA, Microarray

## Abstract

**Background:**

Regardless the regulatory function of microRNAs (miRNA), their differential expression pattern has been used to define miRNA signatures and to disclose disease biomarkers. To address the question of whether patients presenting the different types of diabetes mellitus could be distinguished on the basis of their miRNA and mRNA expression profiling, we obtained peripheral blood mononuclear cell (PBMC) RNAs from 7 type 1 (T1D), 7 type 2 (T2D), and 6 gestational diabetes (GDM) patients, which were hybridized to Agilent miRNA and mRNA microarrays. Data quantification and quality control were obtained using the Feature Extraction software, and data distribution was normalized using quantile function implemented in the Aroma light package. Differentially expressed miRNAs/mRNAs were identified using Rank products, comparing T1DxGDM, T2DxGDM and T1DxT2D. Hierarchical clustering was performed using the average linkage criterion with Pearson uncentered distance as metrics.

**Results:**

The use of the same microarrays platform permitted the identification of sets of shared or specific miRNAs/mRNA interaction for each type of diabetes. Nine miRNAs (hsa-miR-126, hsa-miR-1307, hsa-miR-142-3p, hsa-miR-142-5p, hsa-miR-144, hsa-miR-199a-5p, hsa-miR-27a, hsa-miR-29b, and hsa-miR-342-3p) were shared among T1D, T2D and GDM, and additional specific miRNAs were identified for T1D (20 miRNAs), T2D (14) and GDM (19) patients. ROC curves allowed the identification of specific and relevant (greater AUC values) miRNAs for each type of diabetes, including: i) hsa-miR-1274a, hsa-miR-1274b and hsa-let-7f for T1D; ii) hsa-miR-222, hsa-miR-30e and hsa-miR-140-3p for T2D, and iii) hsa-miR-181a and hsa-miR-1268 for GDM. Many of these miRNAs targeted mRNAs associated with diabetes pathogenesis.

**Conclusions:**

These results indicate that PBMC can be used as reporter cells to characterize the miRNA expression profiling disclosed by the different diabetes mellitus manifestations. Shared miRNAs may characterize diabetes as a metabolic and inflammatory disorder, whereas specific miRNAs may represent biological markers for each type of diabetes, deserving further attention.

## Background

MicroRNAs (miRNAs) are small noncoding single-stranded RNAs of approximately 22 nucleotides, well-conserved among different species. The precursors of miRNAs are transcribed into the nucleus and several steps are required until mature miRNAs can be exported to the cytoplasm and incorporated into the RNA-induced silencing complex (RISC). This process can affect gene expression at posttranslational level, leading to mRNA degradation, translational repression or both. As a consequence, the same mRNA expression can be modulated by one or several miRNAs, while one miRNA can regulate several genes. Much attention has been devoted to the role of miRNAs acting on the posttranscriptional gene control and it has been suggested that they may be used as biomarkers of disorders of varied etiologies [1].

MiRNAs present a tissue-specific expression pattern [2] and are involved in the regulation of several important cellular functions, such as cell cycle regulation, apoptosis, differentiation and maintenance of the immune system cell repertoire [3]. Although many factors are implicated on gene regulation, at least one third of the transcription control may be attributed to the action of miRNAs [4]. The probability of interaction of miRNAs with mRNAs may be evaluated by several algorithms in several databases, including TargetScan, Pictar, miRanda and others [5-7]. The advent of microarray platforms for miRNAs and mRNAs has provided a powerful tool to unveil new mRNA/miRNA interactions. In this context, considering the availability of miRNAs in tissues, plasma or in peripheral blood, aberrant expression of miRNAs has been used as biomarkers for neoplastic and non-neoplastic disorders [8-10].

Diabetes is one of the most prevalent chronic diseases, affecting 6.4% of the world’s adult population [11], and the disease may be classified into three principal types: type 1 (T1D), type 2 (T2D), and gestational diabetes mellitus (GDM). T1D is an autoimmune disease characterized by an inflammatory response, which leads to a progressive destruction of pancreatic beta cells, resulting in deregulation of glucose metabolism and insulin deficiency. T2D is usually a non-insulin-dependent diabetes, generally associated with obesity, caused by deficient insulin secretion by pancreatic beta-cell islets or deficient insulin action in peripheral tissues [12]. GDM has been defined as any degree of glucose intolerance with onset or first recognition during pregnancy, and, in most of cases, after birth delivery occur the stabilization of glucose metabolism and of the oral glucose tolerance test.

Several studies have been conducted to evaluate specifically expressed miRNAs in the diverse types of diabetes, particularly associated with the regulation of insulin production and secretion [13-18], differentiation of human pre-adipocytes [19], and association with T1D pathogenesis [20]. Other studies investigated the involvement of differentially expressed miRNAs in T1D [3] and T2D [21,22]; however, there is no information regarding the comparisons of the major types of diabetes using the same microarray platforms. In the present study, we evaluated the mRNA and miRNA profiles of patients presenting T1D, T2D and GDM, using the same microarray platforms, intending to unveil shared and privately expressed miRNAs.

## Results

**Figure 1:**
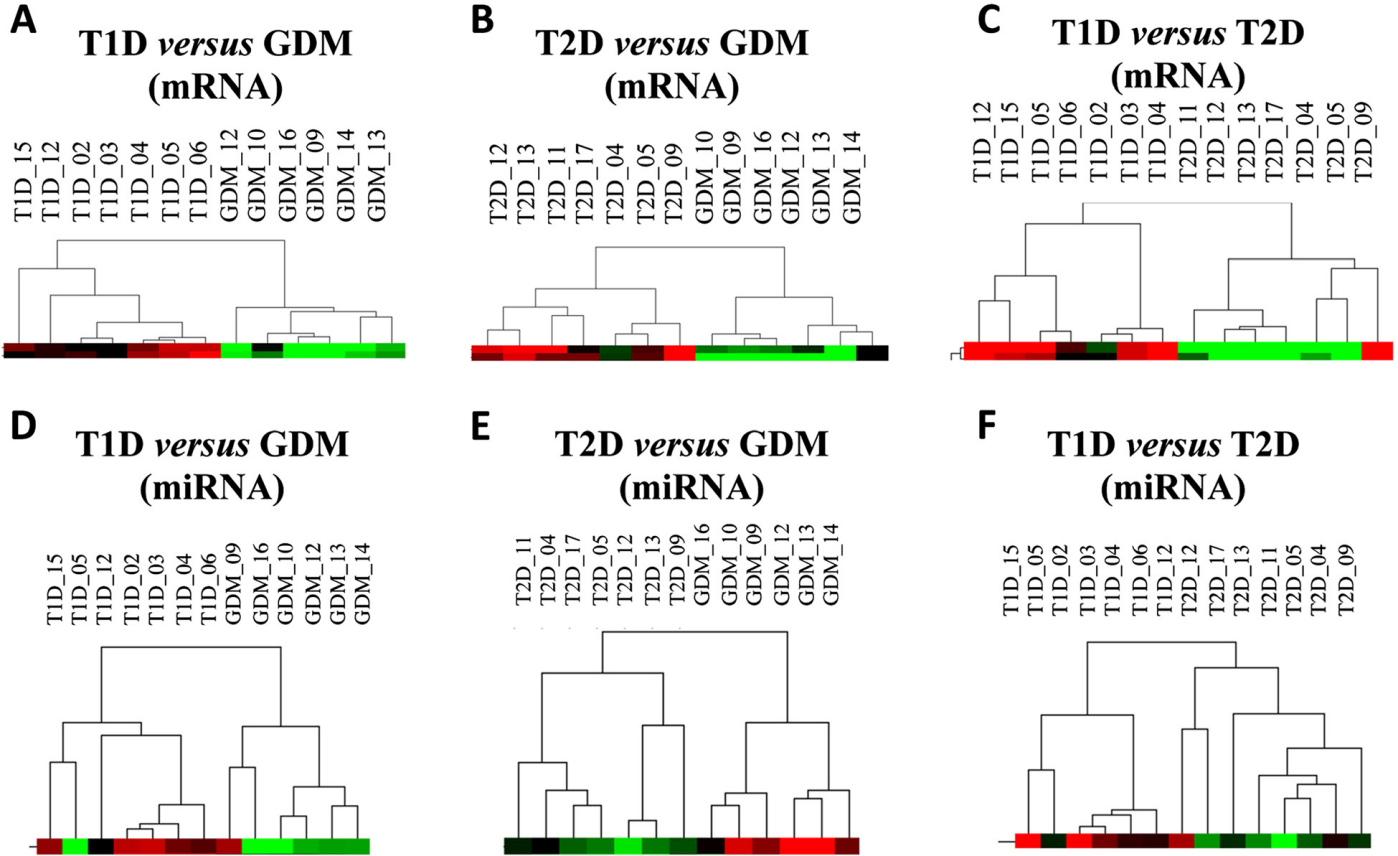
**Hierarchical clustering of mRNA (upper dendrograms) and microRNAs (lower dendrograms).** Clustering analyses refer to the comparisons of the transcript profiles (mRNA and miRNA) between T1D *versus* GDM **(1A and 1D)**, between T2D *versus* GDM **(1B and 1E)**, and between T1D *versus* T2D **(1C and 1F)**. As observed, the mRNA and miRNA profiles were distinct for each type of diabetes.

The differential mRNA/miRNA expression analysis revealed similarities and dissimilarities among the different types of diabetes mellitus. Both mRNA and miRNA analyses were performed comparing two groups; i.e., T1D *versus* GDM, T2G *versus* GDM and T1D *versus* T2D. Statistical analysis of mRNAs by rank products comparing groups of patients yielded 523 differentially expressed transcripts when comparing T1D *versus* GDM, 328 transcripts for T2G *versus* GDM, and 477 for T1D *versus* T2D (*P* ≤ 0.001). Rank products analysis for miRNAs yielded 54 (T1D *versus* GDM), 28 (T2G *versus* GDM) and 31 (T1D *versus* T2D) differentially expressed miRNAs. As seen in Figure 1, the transcript profiles of mRNA and miRNA of patients clearly separated them into distinct clusters.

Overall, the interaction of mRNAs with miRNAs disclosed several predicted interactions, which have been previously described in databanks [23-26]; however, most of these interactions have not been reported in association with diabetes. The comparison between T1D and GDM (523 mRNA and 54 miRNA differentially expressed) revealed 31 predicted interactions, including 21 distinct mRNAs and 13 distinct miRNAs. Among these 21 mRNAs, 8 were downregulated (*ABCA13*, *HTRA3*, *SPTB*, *SLC6A8*, *ANKRD22*, *OXTR*, *GYPA*, and *OR51E2*) and 13 were upregulated (*ACTL6B*, *C1orf87*, *FEZ1*, *IQGAP3*, *DDX3Y*, *EIF1AY*, *ZFY*, *COL13A1*, *HRH4*, *UTY*, *DLG5*, *MS4A2*, and *SLC11A2*). The analysis of miRNAs revealed that 5 out of 13 were down regulated (hsa-miR-636, hsa-miR-939, hsa-miR-720, hsa-miR-595, and hsa-miR-623) and 8 were up regulated (hsa-miR-338-3p, hsa-miR-342-3p, hsa-miR-30b, has-miR-30c, hsa-miR-27a, hsa-miR-27b, hsa-miR-374a, and hsa-miR-92a). These results are shown in Table 1.

**Table 1:** Analyses of interactions of differentially expressed miRNAs and mRNAs, considering T1D* and GDM* patients

**mRNA**	**microRNA**	^**#**^**DIANAmT**	^**#**^**miRanda**	^**#**^**miRDB**	^**#**^**miRWalk**	^**#**^**RNAhybrid**	^**#**^**Targetscan**
*ABCA13*(⬇)	miR-636(⬇)	1	1	1	1	1	1
*HTRA3*(⬇)	miR-636(⬇)	1	1	0	1	1	1
*ACTL6B* (⬆)	miR-939(⬇)	0	1	0	1	0	1
*C1orf87*(⬆)	miR-939(⬇)	1	1	0	1	1	1
*IQGAP3*(⬆)	miR-939(⬇)	1	1	0	1	1	1
*SPTB*(⬆)	miR-939(⬇)	1	1	0	1	1	1
*DDX3Y*(⬆)	miR-338-3p(⬆)	1	1	0	1	0	1
*EIF1AY*(⬆)	miR-338-3p(⬆)	1	1	1	1	1	1
*C1orf87*(⬆)	miR-342-3p(⬆)	0	1	0	1	0	1
*SLC6A8*(⬇)	miR-342-3p(⬆)	1	1	1	1	1	1
*ANKRD22*(⬇)	miR-720(⬇)	0	1	0	1	1	1
*FEZ1*(⬆)	miR-720(⬇)	0	1	1	1	1	1
*COL13A1*(⬆)	miR-30b(⬆)	1	1	0	1	0	1
*EIF1AY*(⬆)	miR-30b(⬆)	1	1	0	1	1	1
*ZFY*(⬆)	miR-30b(⬆)	1	1	0	1	1	1
*COL13A1*(⬆)	miR-30c(⬆)	1	1	0	1	0	1
*EIF1AY*(⬆)	miR-30b(⬆)	1	1	0	1	0	1
*ZNF*(⬆)	miR-30b(⬆)	1	1	0	1	1	1
*OXTR*(⬇)	miR-595(⬇)	1	1	0	1	1	1
*HRH4*(⬆)	miR-623(⬇)	1	1	0	1	1	1
*EIF1AY*(⬆)	miR-27a(⬆)	1	1	0	1	1	1
*EIF1AY*(⬆)	miR-27b(⬆)	1	1	0	1	1	1
*GYPA*(⬇)	miR-347a(⬆)	1	1	1	1	1	1
*ORS51E2*(⬇)	miR-347a(⬆)	1	1	1	1	1	1
*UTY*(⬆)	miR-347a(⬆)	1	1	0	1	1	1
*DDX3Y*(⬆)	miR-92a(⬆)	1	1	0	1	0	1
*DLG5*(⬆)	miR-92a(⬆)	1	1	0	1	0	1
*FEZ1*(⬆)	miR-92a(⬆)	0	1	0	1	1	1
*MS4A2*(⬆)	miR-92a(⬆)	1	1	1	1	1	1
*OXTR*(⬇)	miR-92a(⬆)	1	1	0	1	1	1
*SLC11A2*(⬆)	miR-92a(⬆)	1	1	0	1	0	1

*T1D – type 1 diabetes and GDM – gestational diabetes.

^#^Algorithms for mRNA/miRNA interactions.

Number 1 represents predicted interaction and number 0 indicates non-predicted interaction.

⬆ mRNA or miRNA upregulation and ⬇mRNA or miRNA downregulation.

The comparison T2D *versus* GDM (328 mRNAs and 28 miRNAs differentially expressed) yielded 42 predicted interactions, encompassing 23 transcripts and 17 miRNAs (Table 2). Among the 23 differentially expressed mRNAs, 16 were downregulated (*MMP1*, *RASGEF1B*, *SLC6A8*, *IL1A*, *EGR3*, *OXTR*, *GYPA*, *ORS1E2*, *MMP8*, *SEZ6L2*, *HTRA3*, *C8orf4*, *NR4A1*, *EREG*, *LALBA*, and *CTTN*) and 7 upregulated (*EIF1AY*, *ZFY*, *LPAL2*, *DDX3Y*, *SETD5*, *BAGALNT3*, and *MLYCD*). Among the 17 differentially expressed miRNAs, 6 out of 17 were downregulated (hsa-miR-451, hsa-miR-199a-3p, hsa-miR-595, hsa-miR-1268, hsa-miR-181d, and hsa-miR-486-5p) and 11 were upregulated (hsa-miR-342-3p, hsa-miR-30b, hsa-miR-144, hsa-miR-140-3p, hsa-miR-30e, hsa-miR-142-5p, hsa-miR-378, hsa-miR-181a, hsa-miR-101, hsa-miR-142-3p, and hsa-miR-324-5p) (Table 2).

**Table 2:** Analyses of interactions of differentially expressed miRNAs and mRNAs, considering T2D* and GDM* patients

**mRNA**	**microRNA**	^**#**^**DIANAmT**	^**#**^**miRanda**	^**#**^**miRDB**	^**#**^**miRWalk**	^**#**^**RNAhybrid**	^**#**^**Targetscan**
*MMP1*(⬇)	miR-342-3p(⬆)	1	1	0	1	1	1
*RASGEF1B*(⬇)	miR-342-3p(⬆)	1	1	0	1	1	1
*SLC6A8*(⬇)	miR-342-3p(⬆)	1	1	1	1	1	1
*EIF1AY*(⬆)	miR-30b(⬆)	1	1	0	1	1	1
*IL1A*(⬇)	miR-30b(⬆)	1	1	0	0	0	0
*ZFY*(⬆)	miR-30b(⬆)	1	0	0	0	0	0
*ZFY*(⬆)	miR-144(⬆)	1	1	0	1	1	1
*EGR3*(⬇)	miR-140-3p(⬆)	1	1	0	1	1	1
*LPAL2*(⬆)	miR-140-3p(⬆)	0	0	0	1	1	1
*RASGEF1B*(⬇)	miR-140-3p(⬆)	1	1	0	1	1	1
*DDX3Y*(⬆)	miR-451(⬇)	1	1	0	1	0	1
*OXTR*(⬇)	miR-451(⬇)	1	1	0	1	1	1
*SETD5*(⬆)	miR-451(⬇)	1	1	0	1	1	1
*EIF1AY*(⬆)	miR-30e(⬆)	1	1	1	1	1	1
*IL1A*(⬇)	miR-30e(⬆)	1	1	1	1	1	1
*ZFY*(⬆)	miR-30e(⬆)	1	1	1	1	1	1
*EGR3*(⬇)	miR-142-5p(⬆)	1	1	0	1	1	1
*EIF1AY*(⬆)	miR-142-5p(⬆)	1	1	0	1	1	1
*GYPA*(⬇)	miR-142-5p(⬆)	1	1	0	1	1	1
*OR51E2*(⬇)	miR-142-5p(⬆)	1	1	0	1	1	1
*MMP8*(⬇)	miR-199a-3p(⬇)	0	1	0	1	0	0
*SEZ6L2*(⬇)	miR-199a-3p(⬇)	0	0	0	1	0	1
*B4GALNT3*(⬆)	miR-199a-3p(⬇)	1	1	0	1	1	0
*EIF1AY*(⬆)	miR-199a-3p(⬇)	1	1	0	1	1	0
*OXTR*(⬇)	miR-378(⬆)	1	1	0	1	1	1
*MLYCD*(⬆)	miR-595(⬇)	1	1	0	1	1	0
*OXTR*(⬇)	miR-595(⬇)	1	1	0	1	1	1
*IL1A*(⬇)	miR-181a(⬆)	1	1	1	1	1	1
*OR51E2*(⬇)	miR-181a(⬆)	1	1	0	1	1	1
*SLC6A8*(⬇)	miR-1268(⬇)	0	1	0	1	1	1
*B4GALNT3*(⬆)	miR-1268(⬇)	0	1	0	1	1	1
*GYPA*(⬇)	miR-181d(⬇)	1	1	1	1	1	1
*IL1A*(⬇)	miR-181d(⬇)	1	1	1	1	1	1
*HTRA3*(⬇)	miR-101(⬆)	1	1	1	1	1	1
*C8orf4*(⬇)	miR-101(⬆)	1	1	1	1	1	1
*NR4A1*(⬇)	miR-486-5p(⬇)	1	0	0	1	1	1
*EREG*(⬇)	miR-142-3p(⬆)	1	1	0	1	1	1
*LALBA*(⬇)	miR-142-3p(⬆)	1	1	0	1	1	1
*OR51E2*(⬇)	miR-142-3p(⬆)	1	1	0	1	1	1
*CTTN*(⬇)	miR-142-3p(⬆)	1	1	0	1	1	1
*LALBA*(⬇)	miR-324-5p(⬆)	1	1	0	1	1	1
*ZFY*(⬆)	miR-144(⬆)	1	1	0	1	1	1

*T2D – type 2 diabetes and GDM – gestational diabetes.

^#^Algorithms for mRNA/miRNA interactions.

Number 1 represents predicted interaction and number 0 indicates non-predicted interaction.

⬆ mRNA or miRNA upregulation and ⬇mRNA or miRNA downregulation.

Finally, the comparison T1D *versus* T2D (477 mRNA and 31 miRNA differentially expressed) produced 80 predicted interactions, encompassing 42 mRNAs and 23 miRNAs. Among the differentially expressed mRNAs, 12 were downregulated (*BATF2*, *SPG20*, *ZSWIM3*, *ANKRD22*, *IFNG*, *CA1*, *ADAMTSL4*, *SETD5*, *TRIM36*, *C1orf25*, *C9orf40*, and *PARS2*) and 30 were upregulated (*C1orf87*, *PANX2*, *PDE4D*, *PITPNM2*, *RASGEF1B*, *FBLN2*, *GPR125*, *SNF1LK*, *ABCD3*, *CCL20*, *MPHOSPH6*, *KRT2*, *STK35*, *IL1A*, *CXCL3*, *SLC11A2*, *CRABP1*, *DDX3Y*, *SLC16A3*, *IRS2*, *SNAI1*, *ZNF507*, *ID1*, *SOD2*, *TBKBP1*, *TSPYL5*, *C1orf96*, *DFNB31*, *PER1*, and *EREG*). Regarding miRNAs, 10 were downregulated (hsa-miR-342-3p, hsa-miR-720, hsa-miR-144, hsa-miR-140-3p, hsa-miR-30e, hsa-miR-1260, hsa-miR-1308, hsa-miR-142-5p, hsa-miR-29b, and hsa-miR-142-3p) and 13 were upregulated (hsa-miR-27a, hsa-miR-21, hsa-miR-130a, hsa-miR-150, hsa-miR-223, hsa-miR-451, hsa-let-7a, hsa-let-7e, hsa-let-7f, hsa-let-7 g, hsa-miR-20b, hsa-miR-199a-3p, and hsa-miR-103), yielding 80 interactions shown in Table 3.

**Table 3:** Analyses of interactions of differentially expressed miRNAs and mRNAs, considering T1D* and T2D* patients

**mRNA**	**microRNA**	^**#**^**DIANAmT**	^**#**^**miRanda**	^**#**^**miRDB**	^**#**^**miRWalk**	^**#**^**RNAhybrid**	^**#**^**Targetscan**
*BATF2*(⬇)	miR-342-3p(⬇)	1	1	0	1	1	1
*C1orf87*(⬆)	miR-342-3p(⬇)	0	1	0	1	0	1
*PANX2*(⬆)	miR-342-3p(⬇)	1	1	0	1	1	1
*PDE4D*(⬆)	miR-342-3p(⬇)	0	0	0	1	0	1
*PITPNM2*(⬆)	miR-342-3p(⬇)	1	1	0	1	1	1
*RASGEF1B*(⬆)	miR-342-3p(⬇)	1	1	0	1	1	1
*SPG20*(⬇)	miR-342-3p(⬇)	1	1	0	1	1	1
*ZSWIM3*(⬇)	miR-342-3p(⬇)	1	1	0	1	1	1
*ANKRD22*(⬇)	miR-720(⬇)	0	1	0	1	1	1
*FBLN2*(⬆)	miR-27a(⬆)	1	1	0	1	1	1
*GPR125*(⬆)	miR-27a(⬆)	1	1	0	1	1	1
*IFNG*(⬇)	miR-27a(⬆)	1	1	0	1	1	1
*PITPNM2*(⬆)	miR-27a(⬆)	1	1	0	1	1	1
*SNF1LK*(⬆)	miR-27a(⬆)	1	1	1	1	1	1
*ABCD3*(⬆)	miR-21(⬆)	1	1	1	1	1	1
*CA1*(⬇)	miR-21(⬆)	0/1	0/1	0	1	0	1
*CCL20*(⬆)	miR-21(⬆)	1	1	1	1	1	1
*ABCD3*(⬆)	miR-130a(⬆)	1	1	0	1	1	0
*ABCD3*(⬆)	miR-144(⬇)	0	1	0	0	0	0
*MPHOSPH6*(⬆)	miR-144(⬇)	1	1	1	1	1	1
*KRT2*(⬆)	miR-140-3p(⬇)	1	1	0	1	1	1
*RASGEF1B*(⬆)	miR-140-3p(⬇)	1	1	0	1	1	1
*STK35*(⬆)	miR-140-3p(⬇)	1	1	0	1	1	1
*IL1A*(⬆)	miR-150(⬆)	1	1	1	1	1	1
*ADAMTSL4*(⬇)	miR-223(⬆)	1	0	0	1	1	1
*CXCL3*(⬆)	miR-223(⬆)	0	0	0	1	0	0
*SLC11A2*(⬆)	miR-223(⬆)	1	1	1	1	1	1
*ADAMTSL4*(⬇)	miR-451(⬆)	1	1	0	1	1	0
*CRABP1*(⬆)	miR-451(⬆)	1	1	0	1	1	0
*DDX3Y*(⬆)	miR-451(⬆)	1	1	0	1	0	1
*SETD5*(⬇)	miR-451(⬆)	1	1	0	1	1	1
*SLC16A3*(⬆)	miR-451(⬆)	1	1	0	1	1	1
*ANKRD22*(⬇)	miR-30e(⬇)	1	1	1	1	1	1
*GPR125*(⬆)	miR-30e(⬇)	1	1	1	1	1	1
*IL1A*(⬆)	miR-30e(⬇)	1	1	1	1	1	1
*IRS2*(⬆)	miR-30e(⬇)	1	1	0	1	1	1
*PITPNM2*(⬆)	miR-30e(⬇)	1	1	1	1	1	1
*SETD5*(⬇)	miR-30e(⬇)	0	1	1	1	1	1
*SNAI1*(⬆)	miR-30e(⬇)	1	1	1	1	1	1
*ZNF507*(⬆)	miR-30e(⬇)	1	1	0	1	1	1
*ID1*(⬆)	miR-1260(⬇)	0	1	0	1	1	1
*STK35*(⬆)	miR-1308(⬇)	0	1	0	1	1	1
*SOD2*(⬆)	miR-142-5p(⬇)	1	1	0	1	1	0
*TRIM36*(⬇)	miR-142-5p(⬇)	1	0	0	0	0	1
*C1orf25*(⬇)	let-7a(⬆)	1	1	0	1	1	1
*C9orf40*(⬇)	let-7a(⬆)	0	1	0	1	1	1
*MPHOSPH6*(⬆)	let-7a(⬆)	1	1	0	1	1	1
*PARS2*(⬇)	let-7a(⬆)	1	1	1	1	1	1
*TBKBP1*(⬆)	let-7a(⬆)	1	1	0	1	1	1
*TSPYL5*(⬆)	let-7a(⬆)	0	1	0	1	1	1
*C9orf40*(⬇)	let-7e(⬆)	0	1	0	1	1	1
*IRS2*(⬆)	let-7e(⬆)	1	1	0	1	1	1
*MPHOSPH6*(⬆)	let-7e(⬆)	1	1	0	1	1	1
*PARS2*(⬇)	let-7e(⬆)	1	1	1	1	1	1
*TBKBP1*(⬆)	let-7e(⬆)	1	1	0	1	1	1
*TSPYL5*(⬆)	let-7e(⬆)	0	1	0	1	1	1
*C1orf25*(⬇)	let-7f(⬆)	0	1	0	1	0	1
*C9orf40*(⬇)	let-7f(⬆)	0	1	0	1	1	1
*MPHOSPH6*(⬆)	let-7f(⬆)	1	1	0	1	1	1
*PARS2*(⬇)	let-7f(⬆)	1	1	0	1	0	1
*TBKBP1*(⬆)	let-7f(⬆)	1	1	0	1	0	1
*TSPYL5*(⬆)	let-7f(⬆)	0	1	0	1	1	1
*C1orf25*(⬇)	let-7 g(⬆)	1	1	0	1	1	1
*MPHOSPH6*(⬆)	let-7 g(⬆)	1	1	0	1	1	1
*PARS2*(⬇)	let-7 g(⬆)	1	1	1	1	1	1
*TBKBP1*(⬆)	let-7 g(⬆)	1	1	0	1	1	1
*TSPYL5*(⬆)	let-7 g(⬆)	0	1	0	1	1	1
*C1orf96*(⬆)	miR-29b(⬇)	1	1	1	1	1	1
*DFNB31*(⬆)	miR-29b(⬇)	1	1	0	1	1	1
*ID1*(⬆)	miR-29b(⬇)	1	1	0	1	1	1
*IFNG*(⬇)	miR-29b(⬇)	1	1	0	1	1	1
*PER1*(⬆)	miR-29b(⬇)	1	1	0	1	1	1
*PITPNM2*(⬆)	miR-29b(⬇)	1	1	0	1	1	1
*KRT2*(⬆)	miR-20b(⬆)	1	1	0	1	1	1
*TRIM36*(⬇)	miR-20b(⬆)	1	1	0	1	1	1
*MPHOSPH6*(⬆)	miR-199a-3p(⬆)	1	1	1	1	1	1
*TRIM36*(⬇)	miR-199a-3p(⬆)	1	1	1	1	1	1
*EREG*(⬆)	miR-142-3p(⬇)	1	1	0	1	1	1
*RASGEF1B*(⬆)	miR-103(⬆)	1	1	0	1	1	1
*TBKBP1*(⬆)	miR-103(⬆)	1	1	0	1	0	1

*T1D – type 1 diabetes and T2D – type 2 diabetes.

^#^Algorithms for mRNA/miRNA interactions.

Number 1 represents predicted interaction and number 0 indicates non-predicted interaction.

⬆ mRNA or miRNA upregulation and ⬇mRNA or miRNA downregulation.

**Figure 2:**
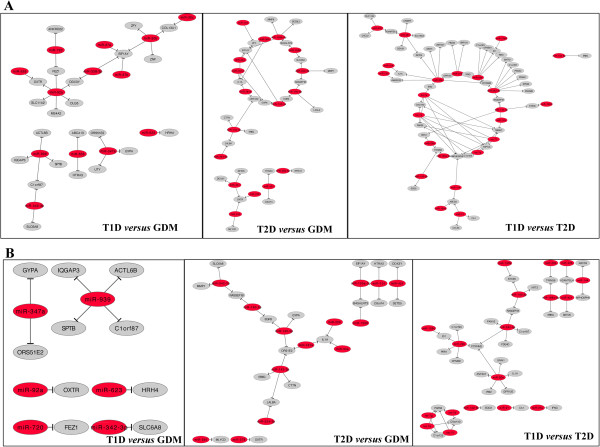
**Networks between miRNAs and mRNAs.** The relationship between miRNAs and mRNAs were evaluated by constructing networks using the Cytoscape software. **(A)** Upper networks show all interactions described in Tables 1, 2 and 3, and **(B)** lower networks show only the negative correlations, i.e., increased miRNA versus decreased mRNA or vice-versa. Red circles represent miRNAs and the grey ones represent mRNA.

As shown in Tables 1, 2 and 3, several types of mRNA/miRNA expected interactions were observed; i. e., induction of a miRNA and repression of an mRNA or vice versa, and also simultaneous induction or repression of both miRNAs and mRNAs. Several predicted interactions were observed, many of them encompassing genes involved in the pathogenesis of diabetes, for instance miR-30e targeting *IL1A* and *IRS2*, miR-181a targeting *IL1A*, miR-223 targeting *SLC11A2*, miR-29b targeting *ID1*, miR-21 targeting *CCL20*, and many others. Figure 2 shows mRNA/miRNA gene networks generated from data observed in Tables 1, 2 and 3 (upper networks show all predicted interactions, and lower networks show only the negative correlations; i. e., increased miRNA versus decreased mRNA or vice-versa).

**Figure 3:**
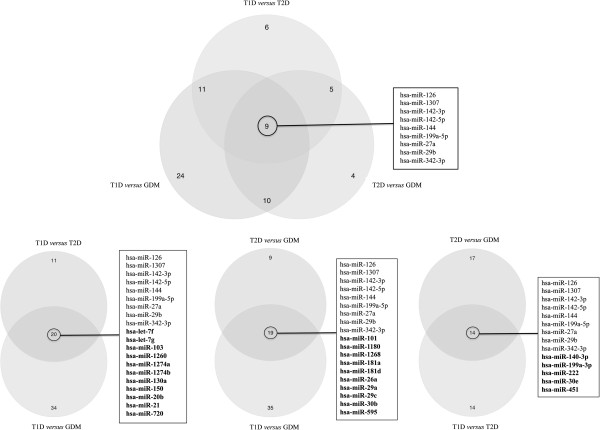
**Venn diagrams showing common and specific microRNAs for the three types of diabetes.** The central intersection of the upper diagram shows the nine shared miRNAs among T1D, T2D and GDM, the upper right intersection shows the 5 miRNAs specific for T2D, the upper left intersection shows the 11 miRNAs specific for T1D, and the middle lower intersection shows the 10 miRNAs specific for GDM patients. Lower Venn diagrams identify specific miRNAs for each type of diabetes (bold letters), as well as the shared ones.

To identify shared and specific miRNAs involved in each type of diabetes, several Venn diagrams were constructed taking into account only the differentially expressed miRNAs (Figure 3). After performing the multiple comparisons between T1D versus T2D, T1D versus GDM and T2D versus GDM, we observed that 9 miRNAs were shared by T1D, T2D and GDM, i.e., hsa-miR-126, hsa-miR-1307, hsa-miR-142-3p, hsa-miR-142-5p, hsa-miR-144, hsa-miR-199a-5p, hsa-miR-27a, hsa-miR-29b, and hsa-miR-342-3p. These miRNAs appear in all intersections of the four Venn diagrams shown in Figure 3. Besides the shared miRNAs, specific miRNAs were identified for each type of diabetes, including: i) T1D: hsa-let-7f, hsa-let-7 g, hsa-miR-103, hsa-miR-1260, hsa-miR-1274a, hsa-miR-1274b, hsa-miR-130a, hsa-miR-150, hsa-miR-20b, hsa-miR-21 and hsa-miR-720, ii) T2D: hsa-miR-140-3p, hsa-miR-199a-3p, hsa-miR-222, hsa-miR-30e and hsa-miR-451, and iii) GDM: hsa-miR-101, hsa-miR-1180, hsa-miR-1268, hsa-miR-181a, hsa-miR-181d, hsa-miR-26a, hsa-miR-29a, hsa-miR-29c, hsa-miR-30b and hsa-miR-595.

**Figure 4:**
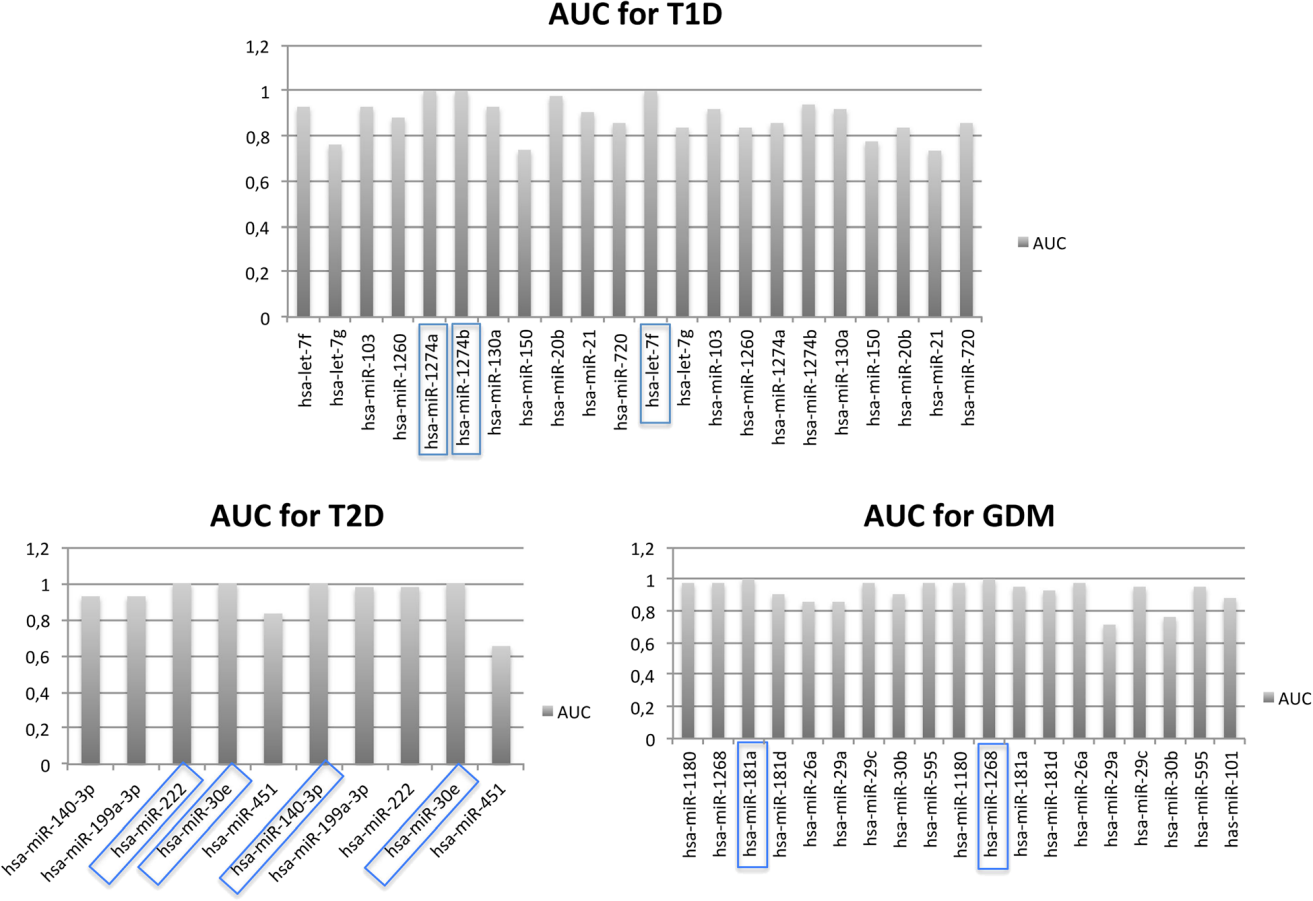
**Identification of most relevant specific miRNAs for each diabetes type.** The values of the area under the curve (AUC) were estimated for all specific miRNAs obtained after the multiple comparisons among the three types of diabetes as shown in Figure 3. MiRNAs exhibiting high AUC values are highlighted within blue rectangles.

The receiver operating characteristic curve (ROC curve) is a diagram to assess sensitivity in function of the specificity. It is usually used in medicine to determine the accuracy of a test to discriminate disease cases from normal cases [27], and to establish a cut-off value for some diagnostic tests. The ROC curve can also be used to compare more than one diagnostic performance from different laboratories [28]. In the present study, the ROC analysis was used to search for potential biomarkers for each type of diabetes. Several ROC curves were constructed for specific miRNAs, considered to be of biological relevance those exhibiting values of area under curve (AUC) greater than 0.8, i.e., high sensibility and high specificity. Accordingly, the hsa-miR-1274a, hsa-miR-1274b and hsa-let-7f presented better results for T1D; hsa-miR-222, hsa-miR-30e and hsa-miR-140-3p for T2D; and hsa-miR-181a and hsa-miR-1268 for GDM patients (Figure 4).

## Discussion

The analysis of miRNA expression profile displayed by the three types of diabetes showed distinct hybridization signatures according to the type of diabetes, in accordance with the results reported by us in a previous study evaluating the mRNA profiling of these patients [29]. The major focuses on the analysis of mRNA/miRNA signatures encompassing T1D, T2D and GDM patients included: i) pinpoint miRNAs shared among the three types of diabetes; ii) select miRNAs specific for each type of diabetes; iii) disclose putative mRNAs targeted by the differentially expressed miRNAs; and iv) identify non-described miRNAs associated with each type of diabetes. All selected miRNAs were detected in at least 3 out of 6 algorithm analyses used in the present study.

MiRNAs play regulatory roles in many biological processes associated with diabetes, including adipocyte differentiation, metabolic integration, insulin resistance and appetite regulation [30]. The role of miRNAs in diabetes has been associated with several pathogenic features, including: i) miR-410, miR-200a and miR-130a regulate secretion of insulin in response to stimulatory levels of glucose, and overexpression of miR-410 enhances the levels of glucose-stimulated insulin secretion [18]; ii) miRNA-143 is upregulated during differentiation of human pre-adipocytes [19]; iii) miR-30d is upregulated in pancreatic beta-cells and collaborates for increasing insulin gene expression [17]; iv) miR-9 acts in the fine-tuning of glucose metabolism [14]; v) miR-375 is involved on the control of insulin gene expression and secretion [13,15]; vi) overexpression of miR-29 represses insulin-stimulated glucose uptake and may result in insulin resistance [31].

Considering that 9 miRNAs were shared among the three types of diabetes, including hsa-miR-126, hsa-miR-144, hsa-miR-27a, hsa-miR-29b, hsa-miR-1307, hsa-miR-142-3p, hsa-miR-142-5p, hsa-miR-199a-5p, and hsa-miR-342-3p, we may suppose that these miRNAs are associated with diabetes *per se*.

MiR-126 has been associated with immune response of adipose tissue and macrophage infiltration into the adipose tissue [32]. It is expressed in endothelial cells and contributes to the endothelial homeostasis and vascular integrity [33]. In diabetic patients, alteration in miR-126 expression may inhibit endothelial progenitor cell proliferation, migration, and may induce apoptosis [34]. High glucose concentration is associated with reduced miR-126 content in endothelial apoptotic bodies [21], and a positive association between miR-126 expression and myocardial infarction has been observed in diabetic patients, probably due to the role of miR-126 on platelet function [35].

MiR-144, miR-27a and miR-29b present a more relevant role on diabetes. MiR-144 targets *IRS1* (insulin receptor substrate-1), a gene highly involved in insulin signaling pathway, and upregulation of this miRNA exhibits a linear relationship with the glycemic status in T2D patients [22]. Thus, the control of miR-144 expression may be a potential therapeutic target for T2D patients, deserving further studies.

MiR-27a together with miR-150, miR-192, miR-320a, and miR-375 regulate several biological events related to the pathogenesis of diabetes [22,36-38]. MiR-27a has been associated with hyperglycemia and metabolic syndrome in T2D patients: i) upregulation of this miRNA has been observed in hyperglycemic rats exhibiting T2D [39]; ii) its expression is associated with the fasting glucose level, suggesting its potential role in the early-phase hyperglycemia [40]; iii) considering that miR-27a has a potential angiogenic function, its downregulation in diabetes patients should reduce the angiogenic potential of endothelial progenitor cells in diabetes [34]. In the present study, miR-27a was highly expressed in T1D followed by T2D and GDM. Although there are no studies evaluating the role of miR-27a in T1D, this miRNA may be involved in shared mechanisms for hyperglycemia control in the major types of diabetes.

Several studies report that the miR-29 family, particularly, miR-29b has a role in diabetes: i) in the T2D rat model (Goto-Kakizaki), overexpression of miR-29 family represses insulin-stimulate glucose uptake, facilitating insulin resistance [31]; ii) miR-29 family members (miR-29a, miR-29b, and miR-29c) are expressed in mouse pancreatic beta-cells, and their expression increases with the age of prediabetic NOD mice [41], contributing to insulin resistance in animal model of diabetes T2D [31]; iii) miR-29 overexpression downregulates glucose-induced insulin secretion in human islet cells [41]; iv) miR-29b is highly expressed in neurons of the retinal ganglion cells of diabetic rats, suggesting a role of miR-29b in the pathogenesis of diabetic retinopathy [42]; v) besides neurons, miR-29b has been associated with progression of renal fibrosis, irrespective of the etiology, including diabetic nephropathy [43]. In concert, these findings indicate that the miR-29b and other members of this family may contribute to diabetes pathogenesis and diabetes microvascular complications.

No association with diabetes has been described for miR-1307, miR-142-3p, miR-142-5p, miR-199a-5p and miR-342-3p; however, miR-142-5p and miR-142-3p have been reported as negative regulators of CD4 T cells, and increased expression of miR-142-5p and miR-142-3p is reported in systemic lupus erythematosus, contributing to reverse T cell hyperactivity, inhibiting antibody production, reducing autoimmune activity and IgG production [44]. Deregulation of some of these miRNAs has been associated with carcinogenesis: i) miR-142-3p with acute myeloid leukemia [45], T-cell leukemogenesis [46], esophageal squamous cell carcionoma [47], and hepatocellular carcinoma [48]; ii) miR-142-5p with T cell chronic lymphocytic leukemia [49] and in pediatric brain tumors [50], and iii) miR-199a-5p with hepatocellular carcinoma [51,52], ovarian cancer [53], and oral squamous cell carcinoma [54]. In addition, miR-342-3p has been associated with cellular proliferation [55], and has been suggested as a potential biomarker for multiple myeloma [56] and prion disease [57]. Interestingly, miR-342-3p was associated with obesity in mice [58], and the possible link between this miRNA and obesity is glucagon, a potential target for miR-342-3p. Furthermore, miR-342-3p is associated with immune response, and it is upregulated in activated B cells [59]. Therefore, it is possible that the role of miR-342-3p in obesity and its association with immune response may link this miRNA with diabetes.

To discuss specific miRNAs for each type of diabetes, we selected only those presenting high values of area under curve (AUC = 1.0) of the ROC curves (Figure 4). Considering that: i) the number of differentially expressed miRNAs is very high, ii) microRNA/mRNA interactions are similarly high, and iii) the number of patients evaluated in each group of diabetes of the present study is low, miRNAs exhibiting high AUC may represent those with greater potential to act as biomarker for larger studies. For T1D, let-7f, miR-1274a and miR-1274b fit this assumption. Overall, let-7 family members act as tumor suppressors [60,61], but the Lin28/let-7 pathway regulates glucose metabolism in different organs, and the treatment with anti-let7 has been suggested as a potential therapy for T2D [62]. The miR-1274a and miR-1274b have never been studied in diabetes, and some authors believe that they represent a fragment of tRNA, without known function [63].

For T2D patients, hsa-miR-140-3p, hsa-miR-222 and hsa-miR-30e were induced and exhibited AUC =1.0. The miR-222 overexpression has also been associated with the development of GDM [64], and is overexpressed in T2D animal models [39] exhibiting obesity [65], and in pancreatic cancer [66]. Mir-140-3p targets the *SIRT1* (sirtuin 1) gene, and reduction of miR-140-3p levels has been associated with calorie restriction [67]. SIRT1 is increased in response to a long-term calorie restriction in a tissue-specific manner [68,69]. Although no association has been reported with diabetes, miR-30e is involved with adipose tissue dysfunction and obesity, as observed for T2D [70]. In addition, miR-30 family members are responsible for expression of mesenchymal proteins during pancreatic fibrosis [71], and miR-30d is upregulated by glucose and increased insulin gene expression, suggesting that miR-30d may be a negative regulator for insulin gene expression [17].

Regarding GDM, two miRNA exhibited AUC =1.0: i) hsa-miR-1268 that was induced and ii) hsa-miR-181a that was repressed in relation to T1D and T2D. The few studies evaluating miR-1268 are inconclusive about its function. On the other hand, miR-181a has been considered to be a link between adipose tissue dysfunction and the development of obesity-associated disorders including T2D [72]. Considering that the overexpression of miR-181a induces hepatic insulin resistance in T2D patients, this miRNA has been suggested as potential diagnostic marker for this type of diabetes [72]; however, miR-181a is also overexpressed in children with new onset T1D [73]. Since the induction of miR-181a has been previously associated with T1D and T2D, and since this miRNA was repressed in GDM when compared to T1D and T2D, miR-181a deserves further studies in GDM in terms of pathophysiology and disease marker.

## Conclusions

The present study revealed shared miRNAs among the major types of diabetes, which were also observed in the MiRDisease database [74], including hsa-miR-29b, miR-142-3p and hsa-miR-142-5p. Many of these shared miRNAs have been associated with metabolic pathways, immunological processes and tumorigenesis. Some of these miRNAs were also shared by other immune-mediated or chronic inflammatory diseases, including psoriasis (hsa-miR-142-3p) and lupus nephritis (hsa-miR-150 and hsa-miR-142-5p).

This is the first miRNA study involving the three major groups of diabetes using the same microarray platform, showing that: i) the miRNA signature of each diabetes subset is distinct, ii) shared miRNAs in the major types of diabetes are associated with biological functions associated with disease pathogenesis, iii) privately expressed miRNAs may be used for further and larger studies as disease biomarkers.

## Methods

### Patients

Peripheral blood mononuclear cells (PBMCs) were obtained from 20 adult patients, i.e., 7 presenting T1D (aged 18–27 years), 7 with T2D (aged 41–61 years) and 6 with GDM (aged 29 to 39 years), followed-up at the Outpatient Clinics of the Division of Endocrinology, Faculty of Medicine of Ribeirao Preto, University of Sao Paulo, Brazil. The demographic, laboratory and treatment features of T1D, T2D, and GDM patients are shown in Table 4. The local Ethics Committee approved the protocol of the study (# 9153/2008) and informed written consents were obtained for all patients. Exclusion criteria included recent episodes of ketoacidosis, active nephropathy, proliferative retinopathy, diabetic foot, high HDL (high-density lipoprotein) levels and diagnosed cardiovascular diseases.

**Table 4:** Demographical, laboratory, and treatment features of type 1 (T1D), type 2 (T2D) and gestational (GDM) diabetic patients

**Subjects**	**Age (years)**	**Gender**	**Insulin regular/NPH**	**Duration of diabetes (years)**	**Blood glucose (mg/dL)**	**Glycated hemoglobin (%)**	**Metformin (mg/day)**	**Duration of pregnancy (weeks)**
T1DM-02	23	M	Yes	13	197	8.3	-	-
T1DM-03	24	M	Yes	6	260	10	-	-
T1DM-04	18	M	Yes	8	23	7.2	-	-
T1DM-05	23	M	Yes	20	178	10.1	-	-
T1DM-06	21	F	Yes	8	223	7.8	-	-
T1DM-12	27	F	Yes	10	257	10.4	-	-
T1DM-15	22	F	Yes	13	143	8.3	-	-
Mean ± SD	22.57 ± 2.76			11.14 ± 4.70	183 ± 82.08	8.87 ± 1.27		
T2DM-04	49	M	No	8	130	7.5	2550	-
T2DM-05	42	M	Yes	11	53	5.1	1700	-
T2DM-09	52	M	No	10	100	6.6	1700	-
T2DM-11	41	F	Yes	3	92	10.2	2550	-
T2DM-12	43	F	No	4	173	10.7	2550	-
T2DM-13	60	F	Yes	20	306	10.9	0	-
T2DM-16	56	F	Yes	20	295	12	2550	-
T2DM-17	61	F	Yes	20	101	7.8	2550	-
Mean ± SD	50.5 ± 8.05			12 ± 7.15	156.25 ± 95.35	8.85 ± 2.43		
GDM-09	33	F	No		80	5	-	35
GDM-10	29	F	Yes		89	5.8	-	37
GDM-12	39	F	No		72	5.7	-	32
GDM-13	29	F	Yes		92	5.2	-	28
GDM-14	30	F	No		82	5	-	34
GDM-16	38	F	Yes		59	9.1	-	34
Mean ± SD	33 ± 4.51				79 ± 12.06	5.96 ± 1.57		33.33 ± 3.07

### Blood collection and RNA samples

Twenty mL of peripheral blood cells were collected and used for isolation of PBMCs by gradient density (Ficoll-Hypaque- Sigma, St. Louis, MO). Total RNA was obtained using the Trizol reagent (Invitrogen, Carlsbad, CA), according to the manufacturer’s instructions. RNA concentrations and ratios were checked with the NanoDrop ND-1000 spectrophotometer (NanoDrop Products, Wilmington, DE) and RNA quality was assessed using the 2100 Bioanalyzer (Agilent Technologies, Santa Clara, CA). All RNA samples exhibited high integrity numbers (RIN) ≥ 9.0.

### Oligo microarrays

Total mRNA was hybridized to one-color Agilent whole human genome 4x44K oligo microarray platforms (Agilent Technologies, Santa Clara, CA), containing ~41.000 transcripts, according to manufacturer’s instructions. MiRNA microarray was performed using Agilent human miRNA platform (v3) 8x15K (Agilent Technologies). After hybridization procedures, the slides were scanned using a Microarray Scanner with Surescan High-Resolution Technology (Agilent Technologies). A complete file providing the hybridization profiles of all samples as well as the quantitative data and experimental conditions are available in the ArrayExpress database [75] through the following account numbers: T1D (E-MEXP-3348), T2D (E-MEXP-3287) and GDM (E-MEXP-3349) for mRNAs, and T1D (E-MEXP-3409), T2D (E-MEXP-3373) and GDM (E-MEXP-3382) for miRNAs. Although these files were recorded into the ArrayExpress database at different dates, the hybridizations were performed at the same period but not in the same date, using the same batch of mRNA and miRNA microarray platforms.

### Data quantification and normalization

Data quantification and quality control were performed using the Feature Extraction (FE) software version 11.0 (Agilent Technologies). Expression data were loaded into R environment [76]. Background adjustment was done by subtracting median background values from the median expression values obtained by FE, and data were subsequently log-transformed. Finally, all the data distribution was normalized by quantile function using the Aroma light package [77,78].

### Rank products statistical analysis

Differentially expressed mRNAs and miRNAs were identified using the Rank products non-parametric method by the R package RankProd [79]. Genes were considered to be of interest if *P*-values and percentage of false positive predictions (pfp) were smaller than 0.001. This test was used to perform paired analysis between T1D *versus* GDM, T2D *versus* GDM and T1D *versus* T2D for mRNA and miRNA microarrays. Hierarchical clustering of differentially expressed genes was performed using average linkage criterion and Pearson uncentered distance as a metric.

### MicroRNA target prediction

We searched for miRNA targets using publicly available predictions. Variables selected for best targets included those present in at least three algorithms, using miRWalk, miRanda, RNAhybrid, and TargetScan [23-26]. The algorithm GenMir++ (Generative model for miRNA regulation) [80] was used to evaluate interactions of the differentially expressed mRNAs and miRNAs, according to their expression profiles, to identify the best candidate interactions using Bayesian inferences. The mRNA/miRNA networks were performed using Cytoscape software [81]. Intending to find association of differentially expressed miRNAs with complex disease, miR2Disease databases [74] were also used.

### ROC curve

Differentially expressed miRNAs were further ranked according to sensitivity and specificity to find the best candidates between T1D vs. T2D, T1D vs. GDM and T2D vs. GDM groups. The criteria for biomarker selection were sensitivity and specificity ≥ 80%, as determined using general logistic models (glm) in the R environment followed by ROC curve analyses using ROCR package [82].

## Abbreviations

miRNA: microRNA; T1D: Type 1 diabetes; T2D: Type 2 diabetes; GDM: Gestational diabetes mellitus; AUC: Area under curve; PBMCs: Peripheral mononuclear cells.

## Competing interests

The authors declare that they have no competing interests.

## Authors’ contributions

CVAC and EAD wrote the paper. CVAC, AFE, GAP and EAD conceived the study and participated in its design and coordination. ETSK, GAP and EAD provided material and analytical tools. AFE, DJX and DMR performed patient selection, sample collection and cell separation. MCFF and MFF were responsible for diabetic patient’s treatment and clinical information data. AFE and DJX performed RNA extraction and sample quality control. CVAC and DJX were responsible for hybridization and acquisition of microarray data. CVAC, AFE, TA and DP performed data analysis. All authors contributed to and approved the final manuscript.
